# Supplementary material for: Fungal Pathogen Emergence: Investigations with an Ustilago maydis × Sporisorium reilianum Hybrid
Source: J Fungi (Basel). 2021 Aug 20;7(8):672. doi: 10.3390/jof7080672 (PMC8400639; doi:10.3390/jof7080672)
Supplement: Supplementary file 1 [file jof-07-00672-s001.zip › jof-1342739-supplementary.pdf]

Supplementary Material

Table S1. Primers used in this study

Primer[a,b]	Nucleotide Sequence (5′ to 3′)[c]	Amplicon Size (bp)[d]	Tm (°C)
**RT-PCR**			
gapdh (UMAG_02491) F1	AGGTCAACATCGGTATCAACG	359/766[d]	64
gapdh (UMAG_02491) R1	GAGATGACGACCTTCTTGG		
gapdh (sr10940.1) F1	AGGTCAACATCGGTATCAACG	359/806[d]	64
gapdh (sr10940.1) R1	GAGATGACGACCTTCTTGG		
Ustilago maydis			
rbf1 (UMAG_03172) F1	AAGGTACGTTCACCGGCATT	194	66
rbf1 (UMAG_03172) R1	AGCCGACTGTTGCATCTCAA		
dik6 (UMAG_03070) F1	AACACAAGCACAACAGGTGC	223	66
dik6 (UMAG_03070) R1	TGCAGTGATAACGCCTGGAG		
kpp6 (UMAG_02331) F1	GCATCGAGTCATCCGTACCC	118	66
kpp6 (UMAG_02331) R1	CTTGAGATCGCGGTGGATCA		
biz1 (UMAG_02549) F1	GGACACATATCACCCCACGG	154	64
biz1 (UMAG_02549) R1	GCGTAGGGTAGGATGGCAAA		
hdp1 (UMAG_12024) F1	TACTCGCACCGGCATTACTC	213	64
hdp1 (UMAG_12024) R1	GATCCCCGCTCCGTTATCTG		
zfp1 (UMAG_01961) F1	CTGGGTTTGGTGATCCGTGA	164	66
zfp1 (UMAG_01961) R1	CTGGCTGAATCACCGTCCTT		
hdp2 (UMAG_04928) F1	ACGTATCGGAGTTCAACGCAT	117	68
hdp2 (UMAG_04928) R1	GTTGGTGTTTTCAGCTCTGCC		
nlt1 (UMAG_04778) F1	GTGGTCGGTGGACAAGCTAT	111	66
nlt1 (UMAG_04778) R1	CAATCCGTATCCCTTGCTGT		
pep1 (UMAG_01987) F1	TCTCTAGCACTCGTCCTGCT	95	64
pep1 (UMAG_01987) R1	GGTTGCGGGTCTACCTTGAA		
pit2 (UMAG_01375) F1	ATCAAGAATCCGCCTGCCAA	109	66
pit2 (UMAG_01375) R1	CTCCGTTAGGATCTGTCGGC		
tin2 (UMAG_05302) F1	GAAGACGGAAAGTGGGCAGA	166	66
tin2 (UMAG_05302) R1	GTGCAGCTGGATCTTCTGGT		
cce1 (UMAG_12197) F1	CGCAGGATGGTAAACCCGAA	162	64
cce1 (UMAG_12197) R1	TTCGGGAGAAGGGGCTAGTT		
Sporisorium reilianum			
rbf1 (Sr14215) F1	GGGTGCTACGCCTTCGTATC	231	68
rbf1 (Sr14215) R1	TCGGATGCACCGGCAGTGTG		
dik6 (Sr14129) F1	ACAGGGTTGGTCAGGAGAGT	101	66
dik6 (Sr14129) R1	GTTGGTCGGGTCCATCTTGA		
kpp6 (Sr13532) F1	ACATCCGATCCATGCCCTTC	141	68
kpp6 (Sr13532) R1	GTGCCAGACACTCCTCCAC		
biz1 (Sr11022) F1	CAGACCTCGTCGTATGCCAA	236	66

biz1 (Sr11022) R1	CCGTTACTCTCGCCAACCAA		
hdp1 (Sr16095) F1	GTCACGAGCCAGAAGGAGAG	89	64
hdp1 (Sr16095) R1	CTCTGCACGTACACCTCGTC		
zfp1 (Sr10717) F1	CGTATTCAACCGACCGCCTT	135	66
zfp1 (Sr10717) R1	CGGGCATACGATCCAAGGAC		
hdp2 (Sr15806) F1	CTGATGGCAAAGGATCGCCT	105	66
hdp2 (Sr15806) R1	TTGCAGACGACTCACCATCC		
nlt1 (sr15650) F1	GTCCGATTCACCTCGATCAC	129	66
nlt1 (sr15650) R1	GCCTTGGATGCTGAGATACC		
pep1 (Sr12947) F1	GCACCACACTCGTTCAAACC	159	68
pep1 (Sr12947) R1	CTTCGACCGACGAGTACCAG		
pit2 (Sr10529) F1	ATGCCAGCCATGCGTCGAT	102	66
pit2 (Sr10529) R1	TCCGAAGTCGAAGAACCAGC		
tin2 (Sr10057) F1	CGCCAGACCAGGATCTGTTT	317	66
tin2 (Sr10057) R1	CCAGACATTCGGGGTTGACA		
cce1 (Sr13927) F1	CCGCTTCTATGGAACCTGCAAA	147	66
cce1 (Sr13927) R1	GCCCAGAACTTGTTGAGCCA		
**RT-qPCR**			
Ustilago maydis			
gapdh (UMAG_02491) F4	CCGTTGTGTCTCAGGACTTC	122	66
gapdh (UMAG_02491) R4	GTAACCCCACTCGTTGTCGT		
rbf1 (UMAG_03172) F8.2	TTCAGACCCGCATTCCAACA	96	66
rbf1 (UMAG_03172) R5.2	GCTCAAGCGTTCGTGGCTAGT		
hdp2 (UMAG_04928) F6	CTGTTCAGGCGTTAGCGAGA	124	66
hdp2 (UMAG_04928) R6	AGCCCAAAGTTGTCAGCAGT		
nlt1 (UMAG_04778) F1	GTGGTCGGTGGACAAGCTAT	111	66
nlt1 (UMAG_04778) R1	CAATCCGTATCCCTTGCTGT		
pit2 (UMAG_01375) F1	ATCAAGAATCCGCCTGCCAA	109	66
pit2 (UMAG_01375) R1	CTCCGTTAGGATCTGTCGGC		
tin2 (UMAG_05302) F5	CCCCTGACAGTCGGAAGGTA	122	64
tin2 (UMAG_05302) R1	GTGCAGCTGGATCTTCTGGT		
cce1 (UMAG_12197) F2	TCTCAGGCGTCTGTAAAGCA	112	64
cce1 (UMAG_12197) R2	CAGAAGATTCGGGAGAAGGGG		
Sporisorium reilianum			
gapdh (sr10940.1) F4	TCGTGGTACGACAACGAGTG	98	66
gapdh (sr10940.1) R4	CAAAGCCTCGAAGCACCG		
rbf1 (Sr14215) F4	CTCTTCTAGCACCAGCAATG	81	64
rbf1 (Sr14215) R4	CTGCTCGAAACGTAGCCCGA		
hdp2 (Sr15806) F2	CTGATGGCAAAGGATCGCCT	105	66
hdp2 (Sr15806) R2	TTGCAGACGACTCACCATCC		
nlt1 (sr15650) F1	GTCCGATTCACCTCGATCAC	129	66
nlt1 (sr15650) R1	GCCTTGGATGCTGAGATACC		
pit2 (Sr10529) F4	TCCTCAGGATCCCGGAAGAC	129	66

pit2 (Sr10529) R4	AGTGGACGTACTGCCAATCG		
tin2 (Sr10057) F1	CTTCGTGGCACCCTCTTCTT	102	64
tin2 (Sr10057) R1	CGAGGAAGAGGCACCGATAC		
cce1 (Sr13927) F2	GAAGCCACCGAGTACGGC	110	66
cce1 (Sr13927) R1	GCCCAGAACTTGTTGAGCCA		
**Expression Vectors**			
UMAG_rbf1			
UMAG_03172 ORF F1	ggaccatggATGGACATCTTGGAACTAGT	1235	60
UMAG_03172 ORF R1	ggagcggccgcCTAGGCGCTCTGCAGTTGAG		
UMAG_03172 F1-seq	ATGGACATCTTGGAACTAGT		
UMAG_03172 R1-seq	AAAGGGACGTTCTTGGCTGT		
UMAG_03172 F2-seq	ACTGGCGAGCGACCTCACGT		
UMAG_03172 F1	AGTACGAGCTACGACGGATTC		
UMAG_03172 R1	GGGTAGGTGTTGGACACATTC		
UMAG_03172 F2	ATGGTAGCGATGCTTGACAA		
UMAG_03172 R2	CTAGTAGCAGGCTGAGTGAGG		
UMAG_03172 F4	AAGGTACGTTCACCGGCATT		
UMAG_03172 R4	AGCCGACTGTTGCATCTCAA		
UMAG_03172 F5	CCGCATTCCAACAGCAACAA		
p123-seq-F1	CTTTTGGGTGCCAGACTTTG		
p123-seq-R1	TGACGTTGATCTCCTTGACG		
UMAG_hdp2			
p123otefhdp2-seq-F1	GCAAGATGATGCGTCCACTA		
p123otefhdp2-seq-F2	AGAAGAATCGCAATCCAACG		
p123otefhdp2-seq-F3	AGGGAGTTCGTGTGCCTCTA		
p123otefhdp2-seq-F4	ATGCACGTTTCAATGCAGAG		
p123otefhdp2-seq-F5	CTTCTTCGGGCATCTTTGAC		
p123otefhdp2-seq-F6	TAGAAGCGACCAGGAGCATC		
p123otefhdp2-seq-F7	GCTGAAAACACCAACGATGA		
p123otefhdp2-seq-R1	GGAAAGACATTGGGAGCGTA		
p123otefhdp2-seq-R2	CGAGTAGAGCCAAAGCATCC		
p123otefhdp2-seq-R3	TTGCTGTGAGCATCCTTGTC		
p123otefhdp2-seq-R4	GCTTGGTGATCCTCGGTAAA		
p123otefhdp2-seq-R5	CGAGCTACCCAAAGCTGAAC		
p123otefhdp2-seq-R6	GTCAGCGTTCGATGACTCAA		
p123otefhdp2-seq-R7	CCAGTATCTACGCCGCTCAT		
UMAG_04928ORFseqF1	ATGTCACAACGACCGCAGAG		
UMAG_04928ORFseqR1	TTAGGGTTCAGCAGCGGCAG		
p123-seq-F1	CTTTTGGGTGCCAGACTTTG		
p123-seq-R1	TGACGTTGATCTCCTTGACG		
UMAG_pit2			
UMAG_01375 ORF F1	ggaggatccATGCTGTTTCGCTCAGCCTT	383	60
UMAG_01375 ORF R1	ggagcggccgcTTATTCCCAGATGACCACAT		

UMAG_01375 F1	ATCAAGAATCCGCCTGCCAA		
UMAG_01375 R1	CTCCGTTAGGATCTGTCGGC		
p123-seq-F1	CTTTTGGGTGCCAGACTTTG		
p123-seq-R1	TGACGTTGATCTCCTTGACG		
	UMAG_cce1		
UMAG_12197 ORF F1	ggaccatggATGCATCGACCAACTAGCCT	410	62
UMAG_12197 ORF R1	ggagcggccgcTTAGTGGTGTACTTGGGACC		
UMAG_12197 F1	CGCAGGATGGTAAACCCGAA		
UMAG_12197 R1-Seq	TTCGTCTCTGCCCTTCTTAT		
p123-seq-F1	CTTTTGGGTGCCAGACTTTG		
p123-seq-R1	TGACGTTGATCTCCTTGACG		
	**PCR Screens**		
Multi-Insert-F	ACCGCTGTTGAGATCCAGTTC	2445	64
Multi-Insert-R	GATGCCGCATAGTTAAGTCCT		
p123-seq-F1	CTTTTGGGTGCCAGACTTTG		
UMAG_03172 R1-seq[b]	AAAGGGACGTTCTTGGCTGT	303	62
p123otefhdp2-seq-R1[b]	GGAAAGACATTGGGAGCGTA	433	62
Um04778 Seq 1R[b]	TGCTGTAGTGAAACGTTGCC	846	62
UMAG_01375 R1[b]	CTCCGTTAGGATCTGTCGGC	474	62
UMAG_12197 R1[b]	TTCGGGAGAAGGGGCTAGTT	496	62

[a] F: forward; R: reverse

[b] Paired with p123-seq-F1

[c] Lower-case letters in primer sequences represent tags containing restriction enzyme sites

[d] Amplicon size containing an intron

Table S2. *U. maydis* characterized genes and *S. reilianum* orthologs selected for investigation in the hybrid

Gene	Function	Virulence of Gene Deletion	Study	*S. reilianum* (% Identity)[a]	
rbf1 *UMAG_03172*	Master regulator that facilitates early sexual and pathogenic development, involved in filament formation, and G2 cell cycle arrest	Severely attenuated	Heimel et al. [1]	*sr14215*	69.9%
biz1 *UMAG_02549*	Involved in appressorium formation, regulates G2 cell cycle arrest, and induces early wave effectors	Severely attenuated	Flor-Parra et al. [2]	*sr11022*	53.4%
hdp1 *UMAG_12024*	Involved in filament formation and regulates G2 cell cycle arrest	No impact	Pothiratana [3]	*sr16095*	47.3%
hdp2 *UMAG_04928*	Involved in appressorium formation and induces early wave effectors	Severely attenuated	Lanver et al. [4]	*sr15806*	57.7%
zfp1 *UMAG_01961*	Influences fungal proliferation and effector expression	Reduced	Cheung et al. (unpublished)	*sr10717*	67.5%

ntl1 *UMAG_04778*	Leaf specific tumor formation and regulates late wave effectors	Severely attenuated	Donaldson et al. [5]; Lanver et al. [6]	*sr15650*	61.6%
tin2 *UMAG_05302*	Cytoplasmic effector, reduces lignin biosynthesis in plant cells, induces anthocyanin production	Reduced	Tanaka et al. [7]	*sr10057*	41.8%
pit2 *UMAG_01375*	Apoplastic effector, an inhibitor of plant cysteine proteases	Severely attenuated	Mueller et al. [8]	*sr10529*	33.7%
pep1 *UMAG_01987*	Apoplastic core effector, an inhibitor of plant peroxidases	Severely attenuated	Doehlemann et al. [9]	*sr12947*	65.2%
cce1 *UMAG_12197*	Apoplastic core effector, required for early infection	Severely attenuated	Seitner et al. [10]	*sr13927*	63.9%
dik6 *UMAG_03070*	7TM domain protein that functions in plant-pathogen interactions	No impact	Weinzierl & Kamper (unpublished)	*sr14129*	92.1%
kpp6 *UMAG_02331*	Involved in appressorium formation and essential for plant penetration	Severely attenuated	Brachmann et al. [11]	*sr13532*	84.8%
clp1 *UMAG_02438*	Required for clamp-like structures, proliferation of filaments, and regulates UPR, *bE/bW*, and *rbf1*	Severely attenuated	Scherer et al. [12]; Heimel et al. [13]	*sr10863*	63.0%

[a] Percent identity calculated by alignment with *U. maydis* ortholog in protein blast
Genes indicated in orange were selected to be overexpressed in the hybrid

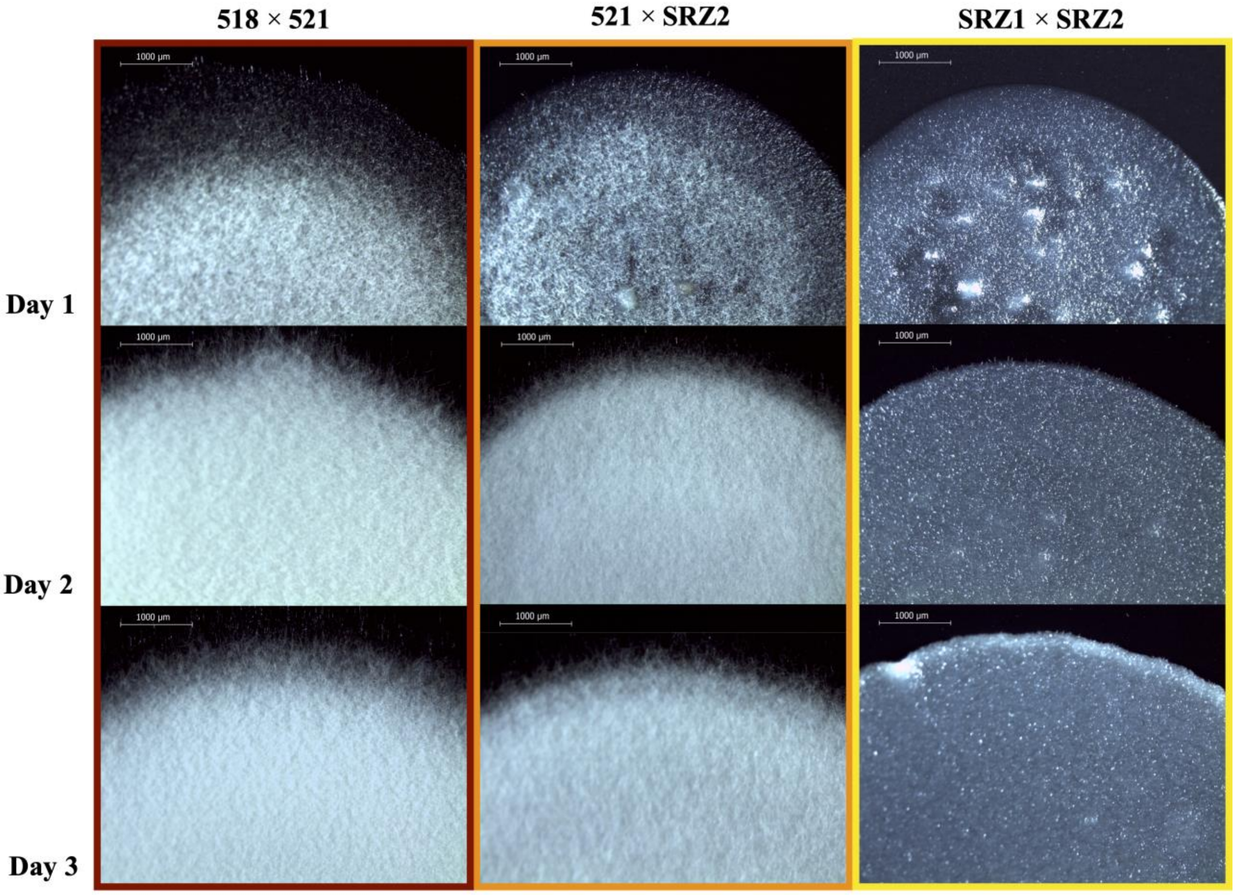

Figure S1. Hybrid dikaryon had slower filament development relative to the *U. maydis* dikaryon. Compatible haploids were pre-mixed and spotted onto PDA + charcoal plates and monitored over three days. Filamentation of the 521 × SRZ2 developed slower than that of 518 × 521. Scale bar, 1000 µm.

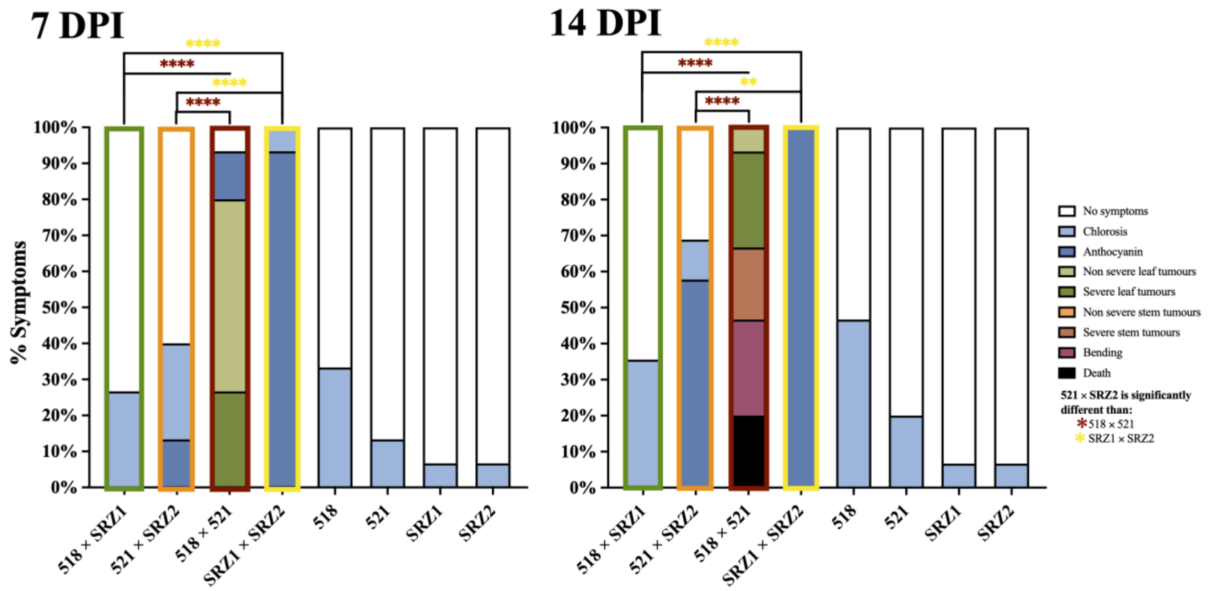

Strains	518 × SRZ1		521 × SRZ2		518 × 521		SRZ1 × SRZ2		518		521		SRZ1		SRZ2	
n	45		45		15		15		15		15		15		15	
	DI	IR%	DI	IR%	DI	IR%	DI	IR%	DI	IR%	DI	IR%	DI	IR%	DI	IR%
7 DPI	0.27	26.70	0.53	40.00	2.93	93.30	1.93	100.00	0.33	33.30	0.13	13.33	0.07	6.67	0.07	6.67
14 DPI	0.36	35.60	1.27	68.90	5.93	100.00	2.00	100.00	0.47	46.67	0.20	20.00	0.07	6.67	0.07	6.67

Figure S2. During infection of maize, 521 × SRZ2 induced minor symptoms of disease at 7 & 14 DPI. Colored bars represent the percentage of plants that developed the symptoms indicated the legend. The table presents the number of plants infected (*n*), disease index (DI), and infection rate (IR). One biological replicate is presented. Statistical differences were calculated using Mann-Whitney U tests (**= $p<0.01$; **** = $p<0.0001$).

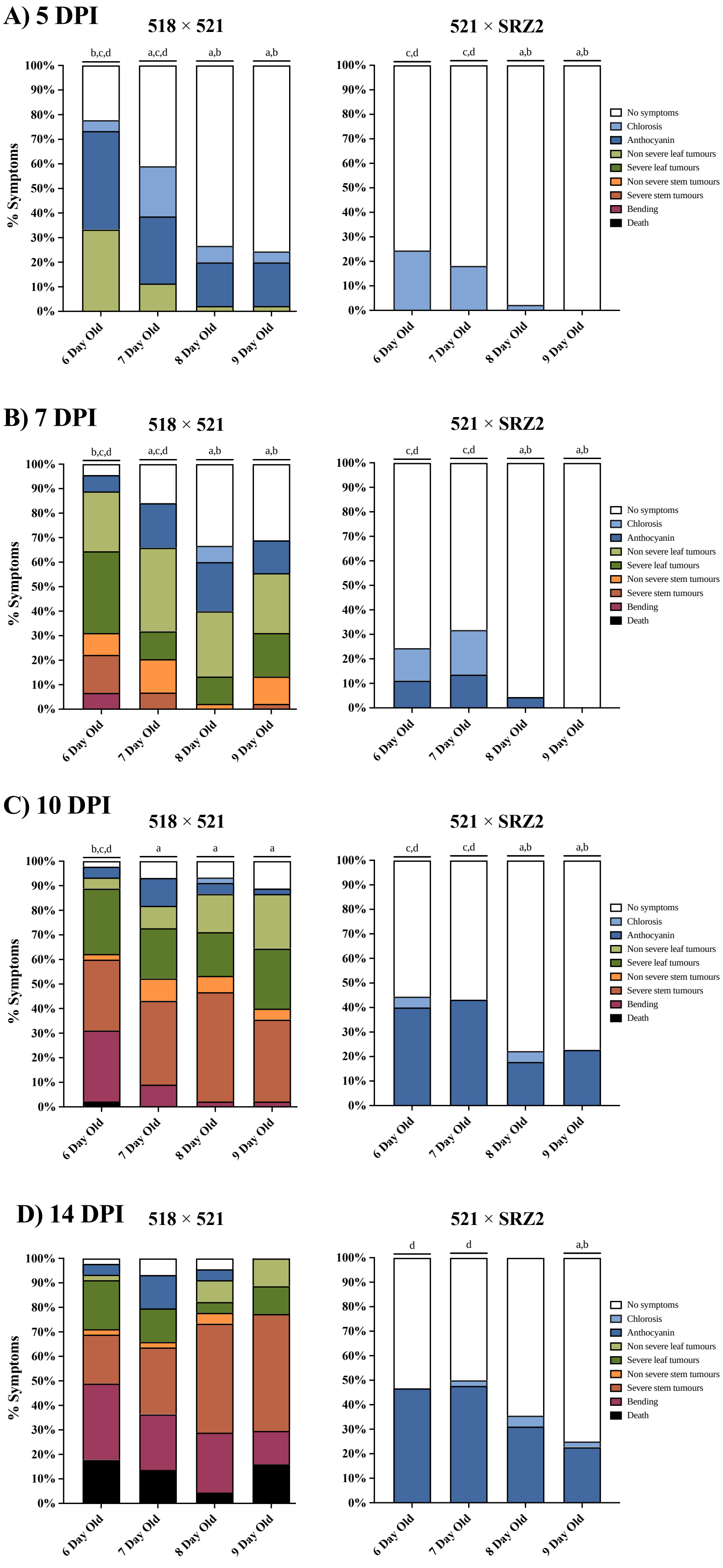

A) 5 DPI
B) 7 DPI
C) 10 DPI
D) 14 DPI
518 × 521
521 × SRZ2
% Symptoms
0%
10%
20%
30%
40%
50%
60%
70%
80%
90%
100%
6 Day Old
7 Day Old
8 Day Old
9 Day Old
b,c,d
a,c,d
a,b
c,d
a
d
No symptoms
Chlorosis
Anthocyanin
Non severe leaf tumours
Severe leaf tumours
Non severe stem tumours
Severe stem tumours
Bending
Death

	Dikaryon	518 × 521				521 × SRZ2			
	Age	6 Day Old	7 Day Old	8 Day Old	9 Day Old	6 Day Old	7 Day Old	8 Day Old	9 Day Old
	n	45	44	45	45	45	44	45	44
5 DPI	DI	1.84	1.09	0.49	0.47	0.24	0.18	0.02	0.00
	IR (%)	77.8	59.1	26.7	24.4	24.4	18.2	2.2	0.00
7 DPI	DI	4.04	2.93	1.82	2.4	0.36	0.45	0.09	0.00
	IR (%)	95.6	84.1	66.7	68.9	24.4	31.8	4.4	0.00
10 DPI	DI	5.33	4.45	4.44	4.07	0.84	0.86	0.4	0.45
	IR (%)	97.8	93.2	93.3	88.9	44.4	43.2	22.2	22.7
14 DPI	DI	5.87	5.25	5.49	5.8	0.93	0.98	0.67	0.48
	IR (%)	97.8	93.3	95.6	100	46.7	51.1	35.6	26.7

Figure S3. The ability of 521 × SRZ2 to infect was reduced when older *Z. mays* seedlings were inoculated. A) 5 DPI, B) 7 DPI, C) 10 DPI, and D) 14 DPI. Colored bars represent the percentage of plants that developed symptoms, as indicated in the legend. The table presents the number of plants infected (*n*), disease index (DI), and infection rate (IR). Statistical differences were calculated using Mann-Whitney U tests at p<0.05 (a, b, c, and d indicate significant differences from 6-, 7-, 8-, and 9-day old seedling inoculations, respectively). One experiment is represented.

A)

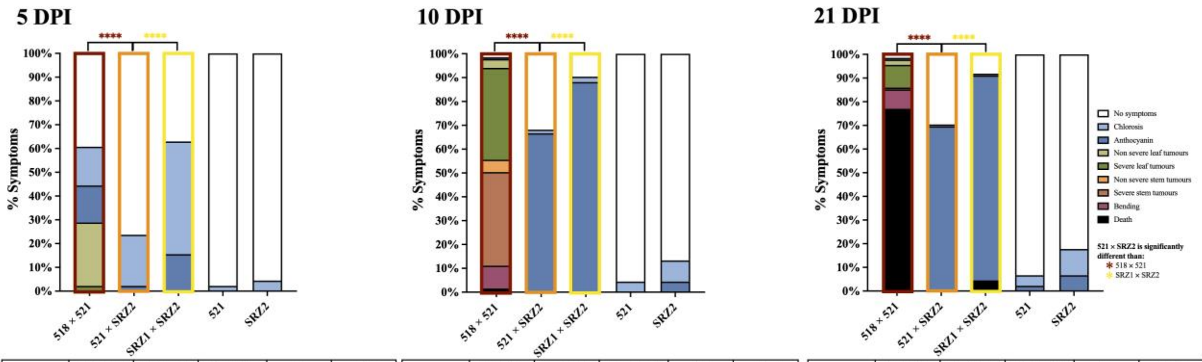

	518 × 521	521 × SRZ2	SRZ1 × SRZ2	521	SRZ2
n	135	135	135	45	45
DI	1.36	0.26	0.79	0.022	0.044
IR (%)	60.7	23.7	63	2.2	4.4

	518 × 521	521 × SRZ2	SRZ1 × SRZ2	521	SRZ2
n	135	135	135	45	45
DI	3.52	0.83	1.4	0.044	0.089
IR (%)	91.1	48.1	83.7	4.4	8.9

	518 × 521	521 × SRZ2	SRZ1 × SRZ2	521	SRZ2
n	135	135	135	45	45
DI	7.20	1.40	2.10	0.089	0.24
IR (%)	98.5	70.4	91.9	91.9	17.8

B)

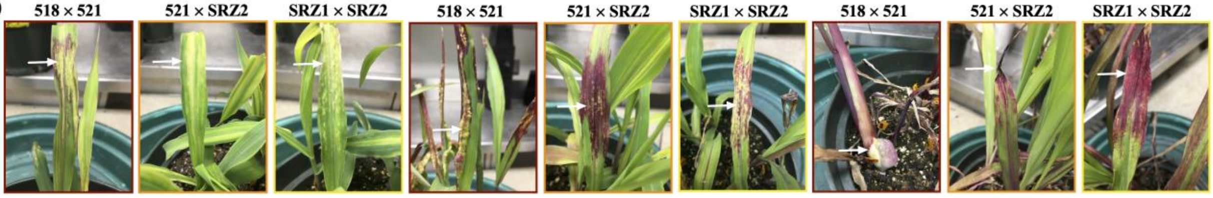

Figure S4. The hybrid was less virulent than the parental dikaryons at 5, 10, & 21 DPI. A) Colored bars represent the percentage of plants that developed symptoms, as indicated in the legend. Statistical differences were calculated using Mann-Whitney U tests (**** = p<0.0001). The table presents the number of plants infected (*n*), disease index (DI), and infection rate (IR). Three separate experiments were conducted, and combined results are presented. B) Representative symptoms, indicated by the arrows, for 518 × 521 infections are anthocyanin and chlorosis at 5 DPI, leaf tumors at 10 DPI, and stem tumors at 21 DPI, for 521 × SRZ2 infections are chlorosis at 5 DPI and anthocyanin at 10 & 21 DPI, and for SRZ1 × SRZ2 infections are chlorosis at 5 DPI, anthocyanin and chlorosis at 10 DPI, and anthocyanin at 21 DPI.

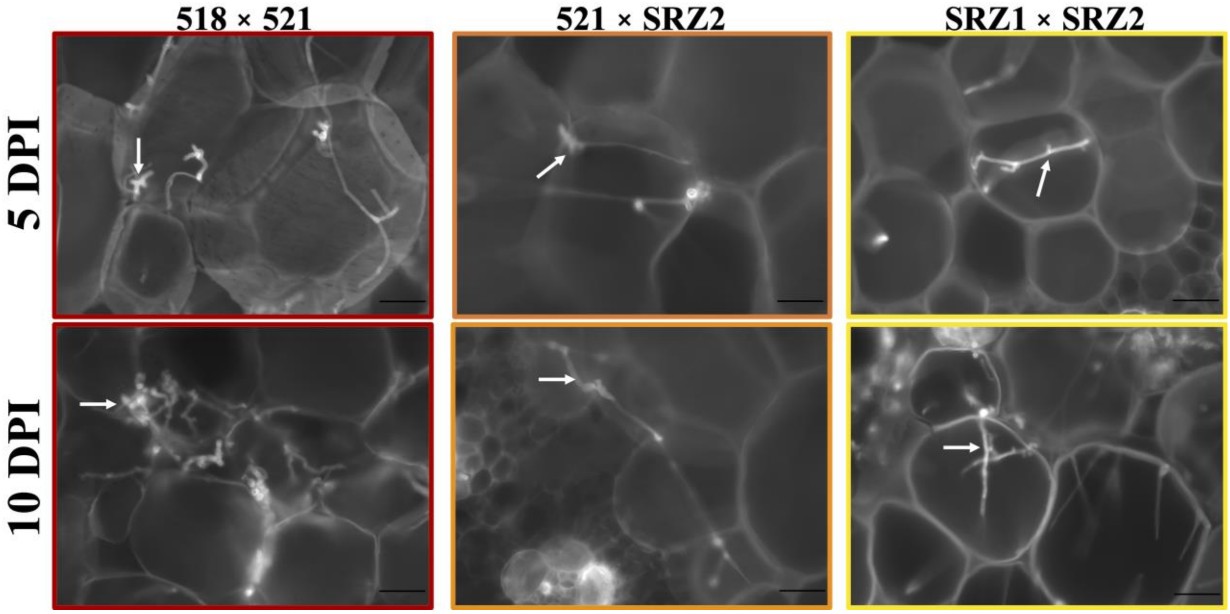

Figure S5. Hyphal growth during infections with the hybrid was reduced compared to parental dikaryon infections at 5 & 10 DPI. Infected plant leaves cross sectioned and were stained with Fungi-Fluor and viewed under Zeiss microscope at 400×. White arrows indicate hyphal growth through plant cells and blue arrows indicate hyphal branching. Scale bar, 20 μm.

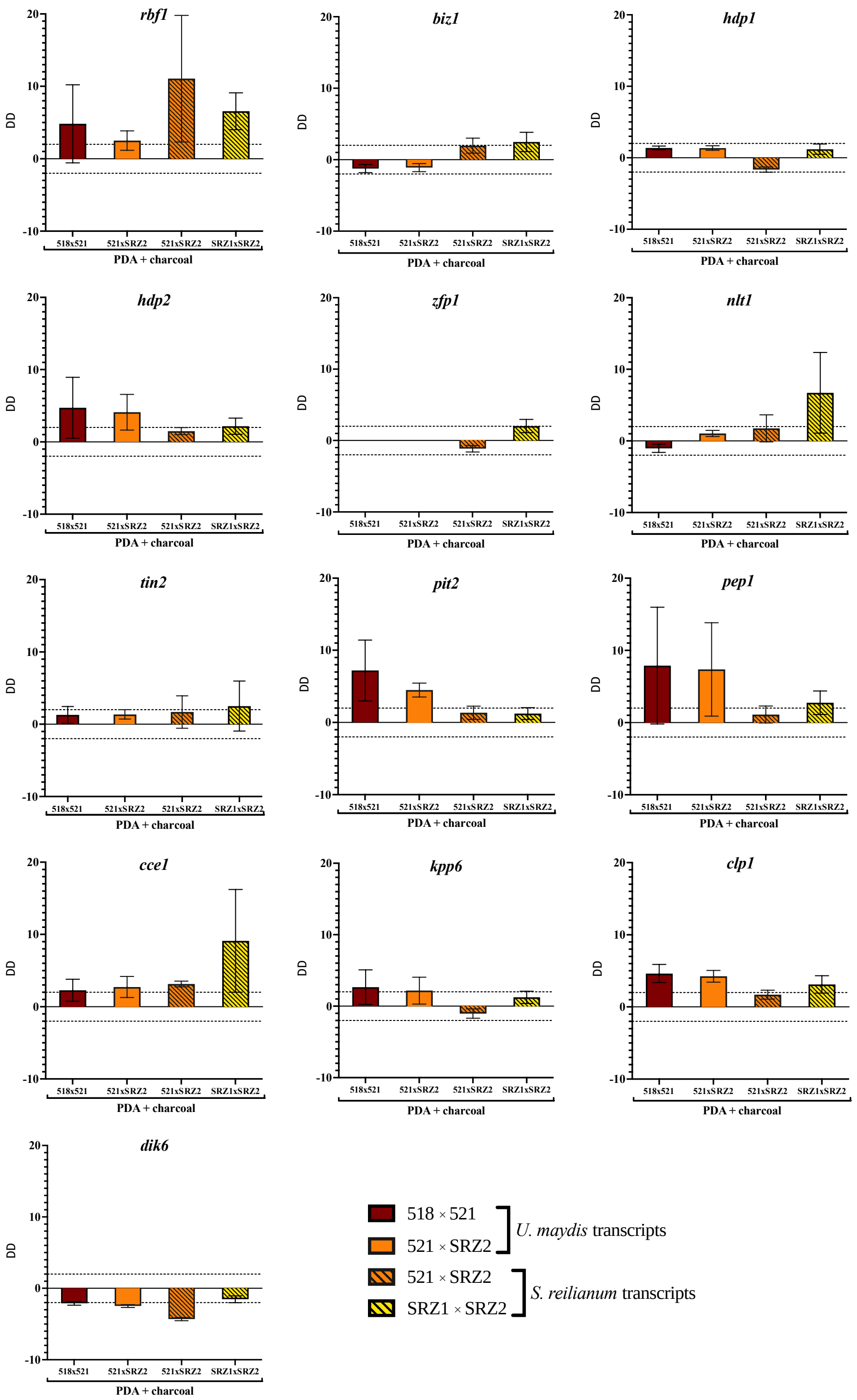

Figure S6. Transcript levels of key *U. maydis* virulence genes and their putative *S. reilianum* orthologues in the hybrid were similar to those of the parental dikaryons during growth on plates. RNA was isolated from mycelia harvested after three days growth on PDA + charcoal plates and used in RT-PCR. Band intensity was quantified in Image Studio Lite. Transcript intensity was normalized to the housekeeping gene (*gapdh*) and fold change was compared non-pathogenic haploids (521 or SRZ2) for reference. Three biological replicates were averaged with standard deviation (error bars). Transcripts with less than a two-fold change in either direction, indicated by the dashed lines at 2 and -2 on the y-axis, were considered unchanged.

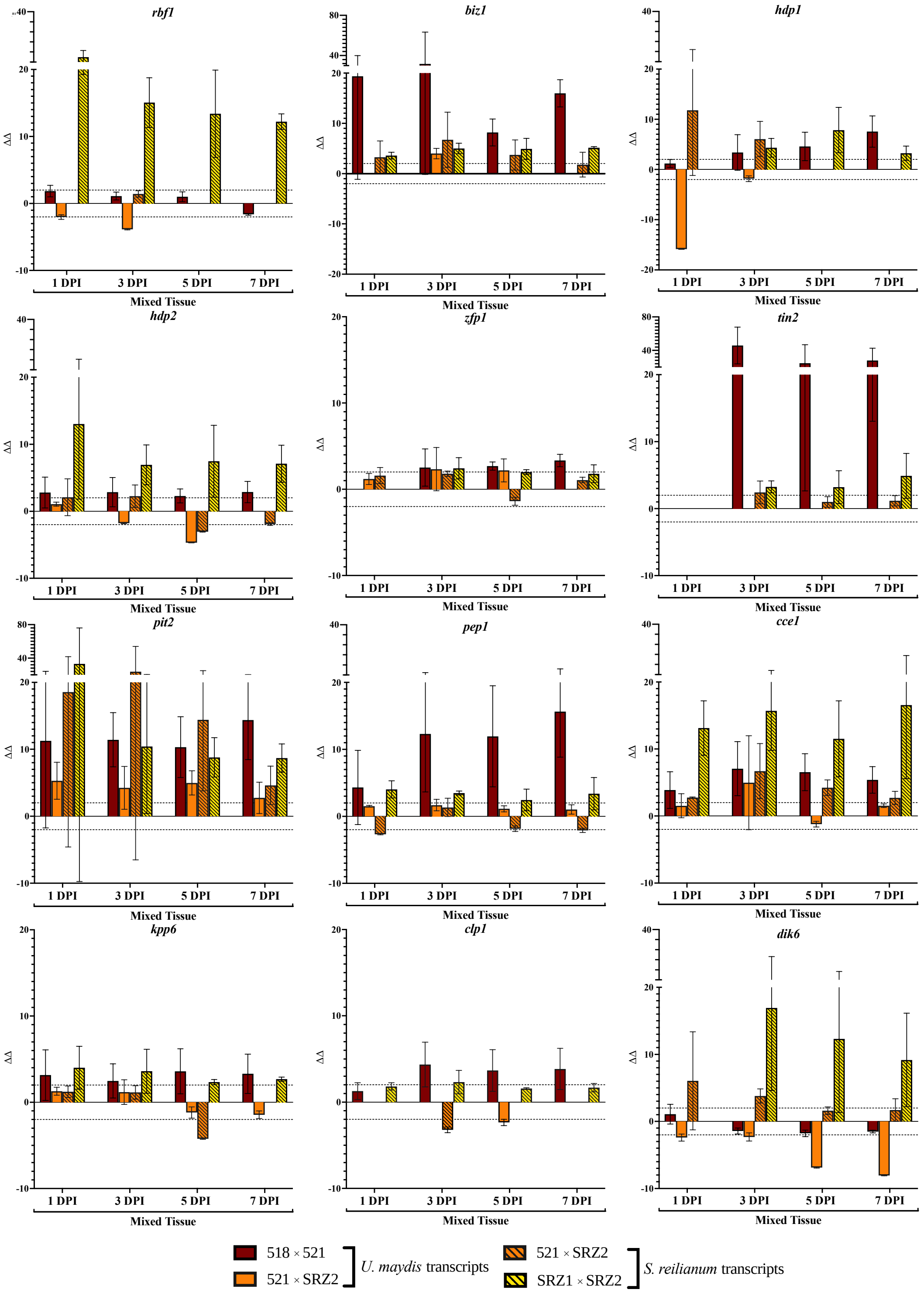

Figure S7. Transcript levels of key *U. maydis* virulence genes and their putative *S. reilianum* orthologues during the hybrid infection had reduced levels and altered expression patterns relative to those in parental dikaryon infections. RNA was isolated from infected plant tissue harvested at 1, 3, 5, & 7 DPI and used in RT-PCR. Band intensity was quantified in Image Studio Lite and transcript intensity was normalized to the housekeeping gene (*gapdh*) and fold change was compared to the axenically grown haploid (521 or SRZ2) for reference. Three biological replicates were averaged with standard deviation reported (error bars). Transcripts with less than a two-fold change in either direction, indicated by the lines at 2 and -2 on the y-axis, were considered unchanged.

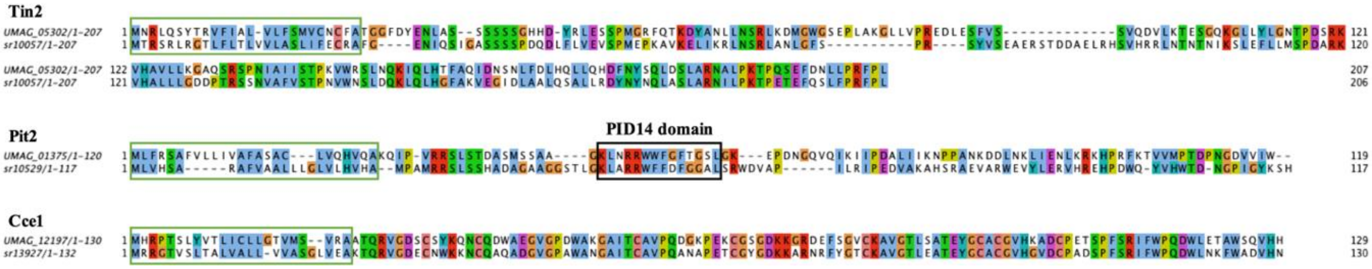

Figure S8. Signal peptides are not conserved between the *U. maydis* effectors and the proposed *S. reilianum* orthologs. *S. reilianum* predicted orthologs were identified using the RBH protein blast and aligned to the corresponding *U. maydis* proteins using MAFFT and visualized in Jalview with a ClustalX color scheme. Signal peptides identified using SignalP are indicated by a green box and functional domains are indicated by a black box. Pit2 contains a PID14 domain.

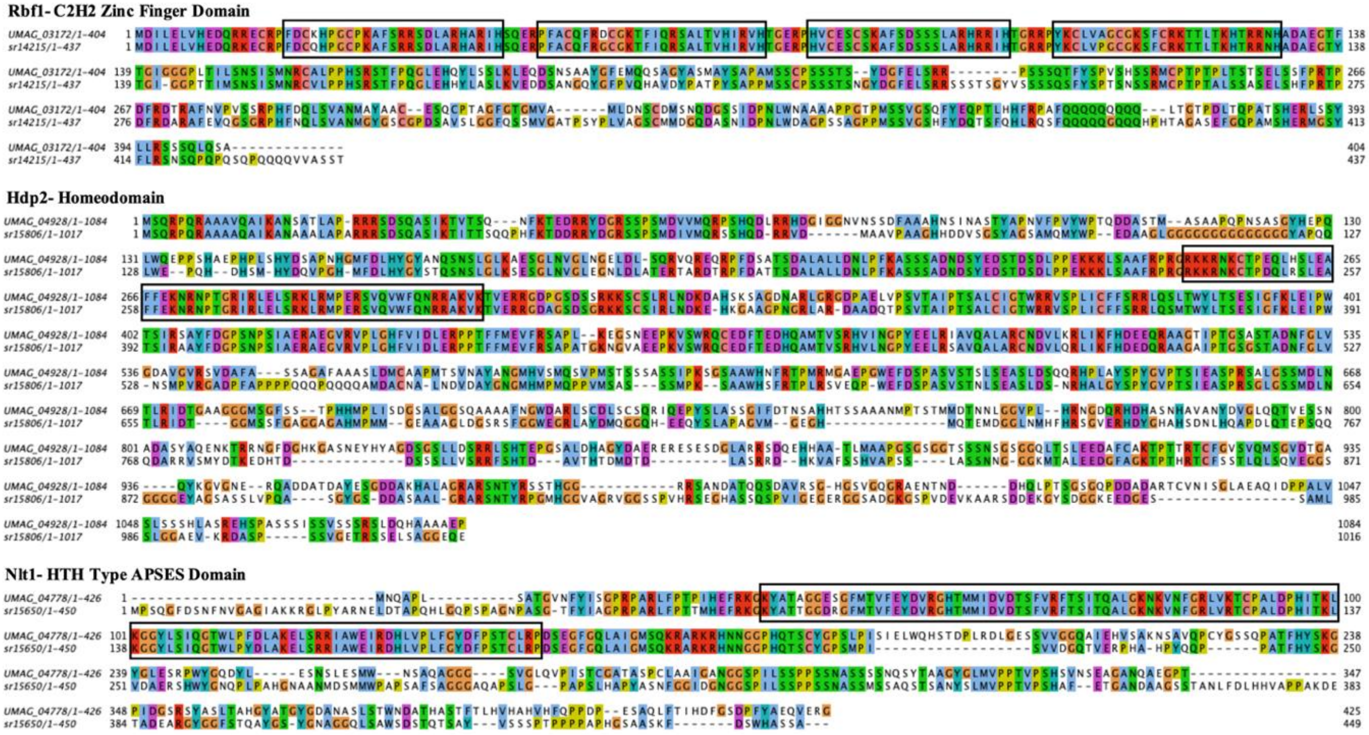

Figure S9. Transcription factor domains are conserved between *U. maydis* and the proposed *S. reilianum* orthologs. *S. reilianum* predicted orthologs were identified using the RBH protein blast and aligned to the corresponding *U. maydis* proteins using MAFFT and visualized in Jalview with a ClustalX color scheme. Domains, indicated by a black box, were identified through PROSITE and PFAM. Rbf1 contains the C2H2 zinc finger domains, Hdp2 contains a homeodomain, and Nlt1 contains an HTH APSES domain.

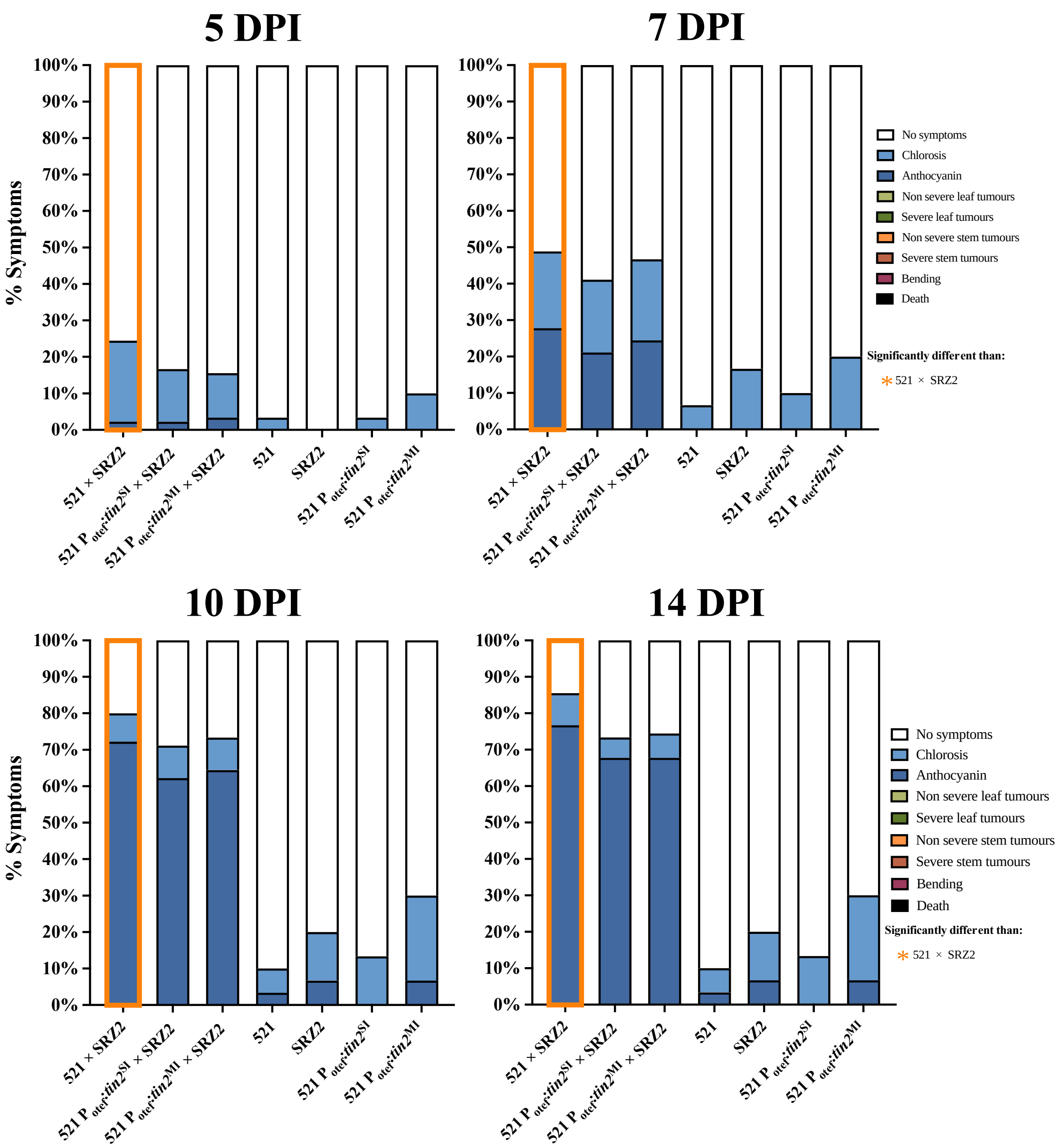

Strains	521 × SRZ2		521 P_{otef}:*tin2*^{SI} × SRZ2		521 P_{otef}:*tin2*^{MI} × SRZ2		521		SRZ2		521 P_{otef}:*tin2*^{SI}		521 P_{otef}:*tin2*^{MI}	
n	90		90		90		45		45		45		45	
	DI	IR%	DI	IR%	DI	IR%	DI	IR%	DI	IR%	DI	IR%	DI	IR%
5 DPI	0.27	24.4	0.19	16.7	0.19	16.7	0.03	3.3	0.00	0.0	0.03	3.3	0.10	10.0
7 DPI	0.77	48.9	0.62	41.1	0.71	46.7	0.07	6.7	0.17	16.7	0.10	10.0	0.20	20.0
10 DPI	1.52	80.0	1.33	71.1	1.38	73.3	0.13	10.0	0.27	20.0	0.13	13.3	0.37	30.0
14 DPI	1.62	85.6	1.41	73.3	1.42	74.4	0.13	10.0	0.27	20.0	0.13	13.3	0.37	30.0

Figure S10. Constitutively expressing *tin2* in the hybrid had no observable impact on pathogenic development at 5, 7, 10, & 14 DPI. Symptoms were scored after infection of seven-day old *Z. mays* seedlings. Colored bars represent the percentage of plants that developed symptoms, as indicated in the legend. Mann-Whitney U tests found no significant difference. Table indicates the number of plants infected (*n*), disease index (DI), and infection rate (IR). Two separate experiments were conducted, and combined results are presented.

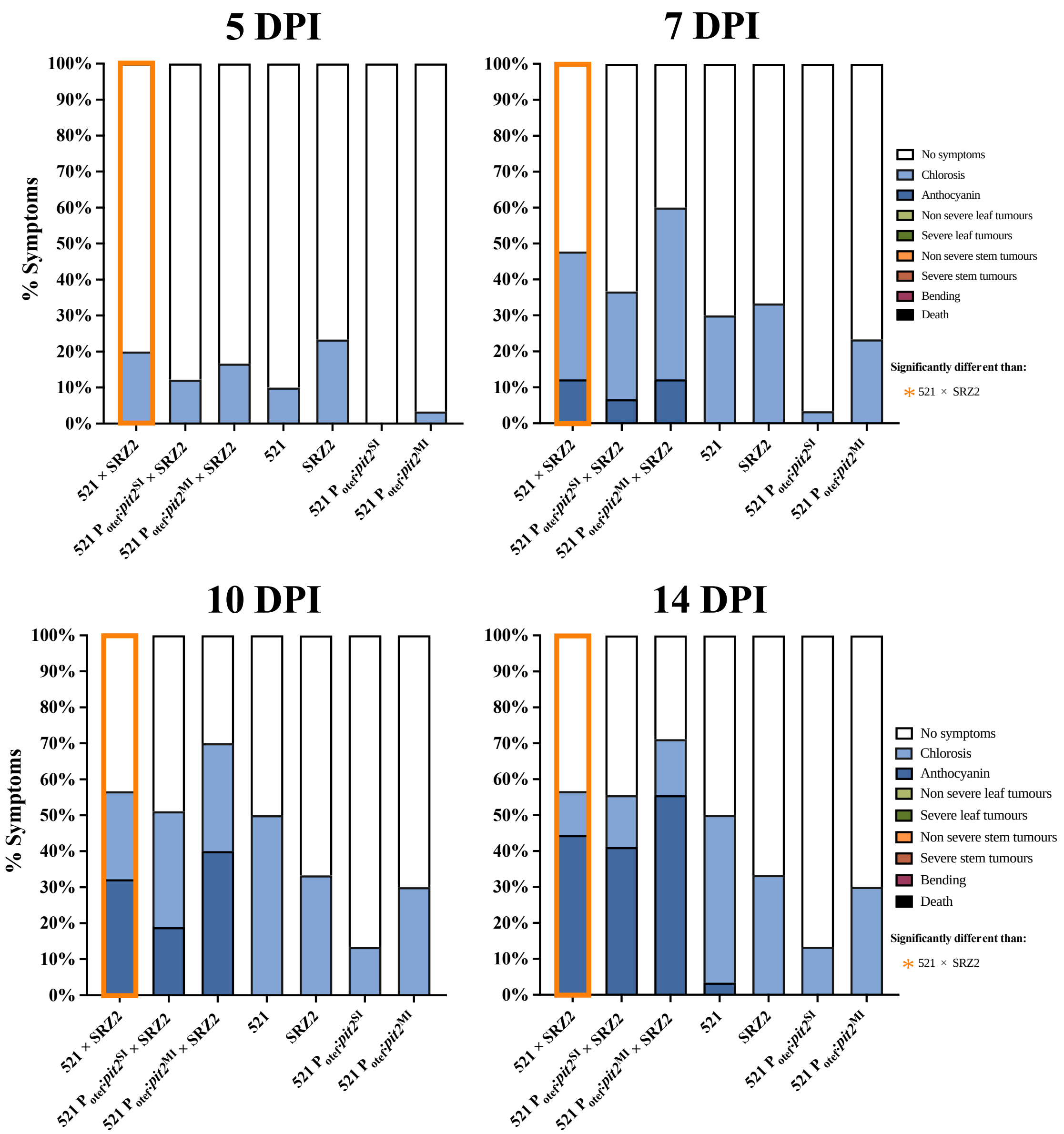

Strains	521 × SRZ2		521 P_{otef}:*pit2*^{SI} × SRZ2		521 P_{otef}:*pit2*^{MI} × SRZ2		521		SRZ2		521 P_{otef}:*pit2*^{SI}		521 P_{otef}:*pit2*^{MI}	
n	90		90		90		45		45		45		45	
	DI	IR%	DI	IR%	DI	IR%	DI	IR%	DI	IR%	DI	IR%	DI	IR%
5 DPI	0.20	20.0	0.12	12.2	0.17	16.7	0.10	10.0	0.23	23.0	0.00	0.0	0.03	3.3
7 DPI	0.60	47.8	0.43	36.7	0.72	60.0	0.33	30.0	0.33	33.3	0.03	3.3	0.23	23.3
10 DPI	0.89	56.7	0.70	51.1	1.10	70.0	0.50	50.0	0.33	33.3	0.13	13.3	0.30	30.0
14 DPI	1.01	56.7	0.97	55.6	1.27	71.1	0.53	50.0	0.33	33.3	0.13	13.3	0.30	30.0

Figure S11. Constitutively expressing *pit2* in the hybrid had a slightly variable impact on infection rate at 5, 7, 10, & 14 DPI. Symptoms were scored after infection of seven-day old *Z. mays* seedlings. Colored bars represent the percentage of plants that developed symptoms, as indicated in the legend. Mann-Whitney U tests found no significant difference. Table indicates the number of plants infected (*n*), disease index (DI), and infection rate (IR). Two separate experiments were conducted, and combined results are presented.

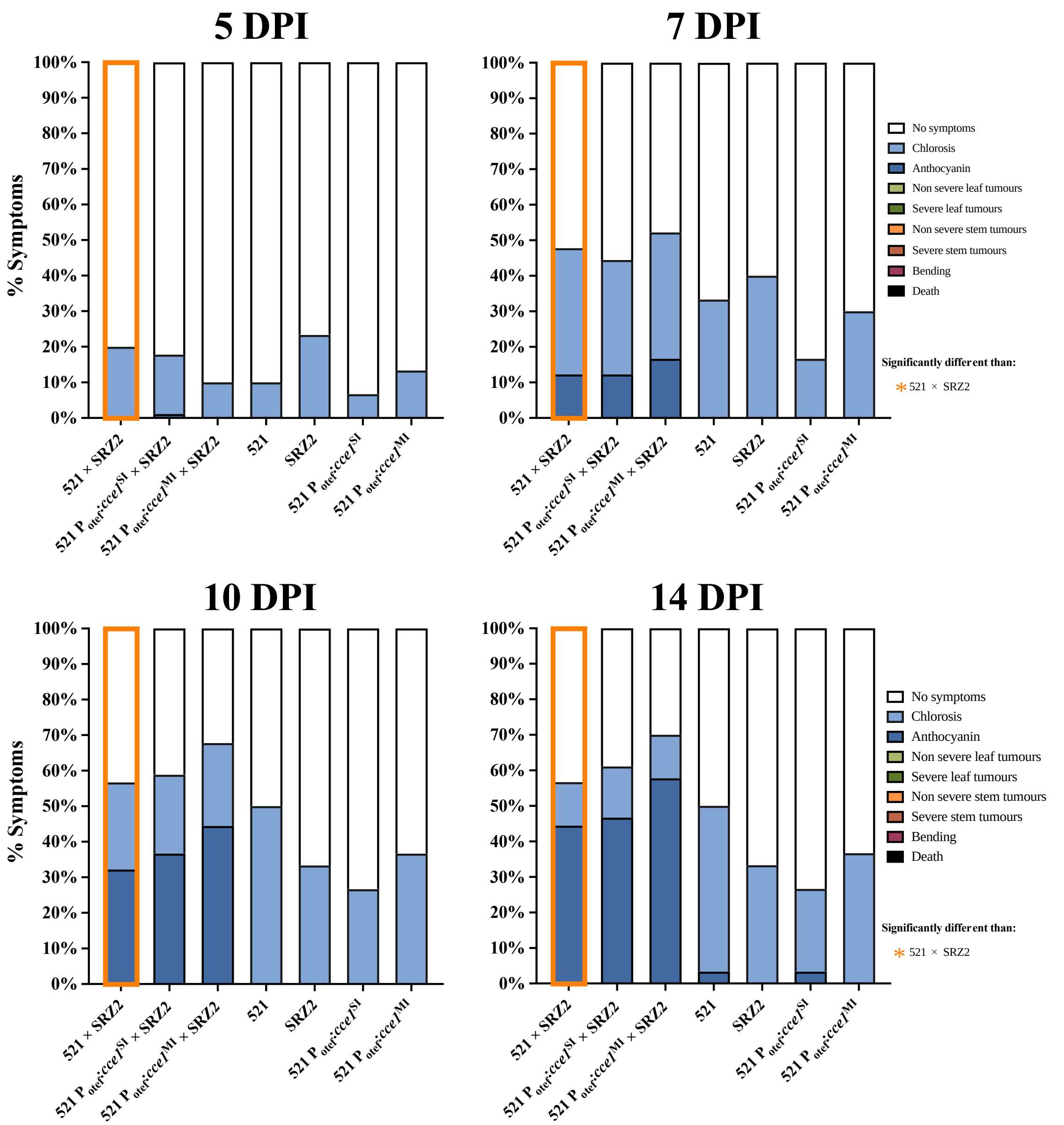

Strains	521 × SRZ2		521 P_{otef}:*cce1*^{SI} × SRZ2		521 P_{otef}:*cce1*^{MI} × SRZ2		521		SRZ2		521 P_{otef}:*cce1*^{SI}		521 P_{otef}:*cce1*^{MI}	
n	90		90		90		45		45		45		45	
	DI	IR%	DI	IR%	DI	IR%	DI	IR%	DI	IR%	DI	IR%	DI	IR%
5 DPI	0.20	20.0	0.19	17.8	0.10	10.0	0.10	10.0	0.23	23.0	0.07	6.7	0.13	13.3
7 DPI	0.60	47.8	0.57	44.4	0.69	52.2	0.33	30.0	0.40	40.0	0.17	16.7	0.30	30.0
10 DPI	0.89	56.7	0.96	58.9	1.12	67.8	0.50	50.0	0.33	33.3	0.27	26.7	0.37	36.7
14 DPI	1.01	56.7	1.08	61.1	1.28	70.0	0.53	50.0	0.33	33.3	0.30	26.7	0.37	36.7

Figure S12. Constitutively expressing *cce1* slightly increased the infection rate compared to the unaltered hybrid at 5, 7, 10, & 14 DPI. Symptoms were scored after infection of seven-day old *Z. mays* seedlings. Colored bars represent the percentage of plants that developed symptoms, as indicated in the legend. Mann-Whitney U tests found no significant difference. Table indicates the number of plants infected (*n*), disease index (DI), and infection rate (IR). Two separate experiments were conducted, and combined results are presented.

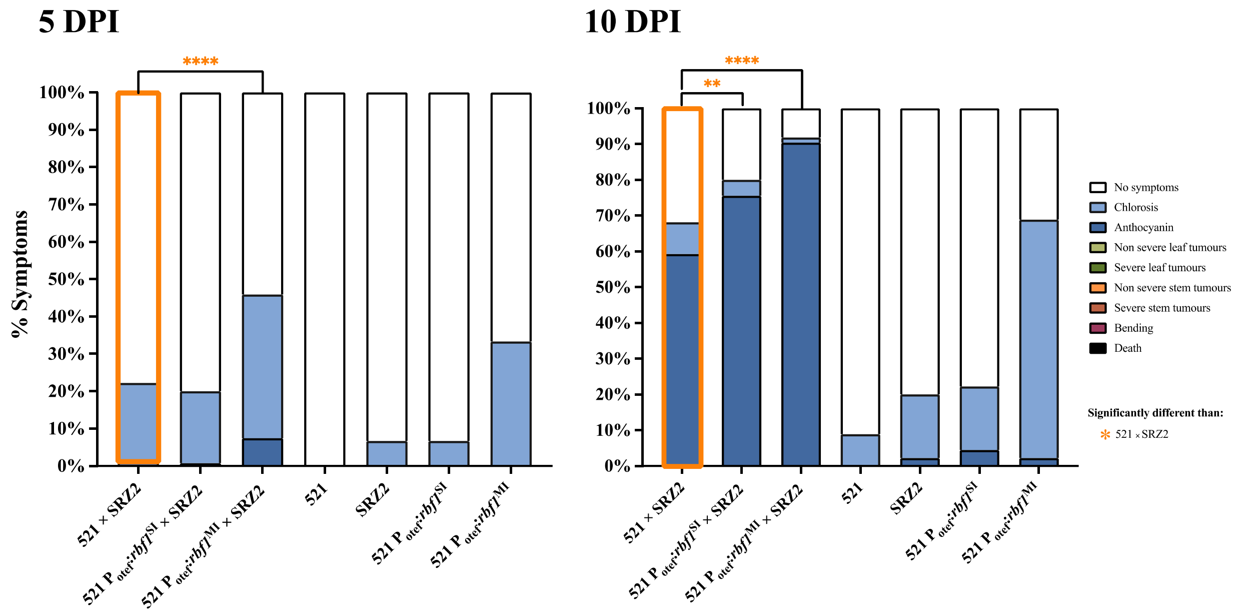

Strains	521 × SRZ2		521 P_{otef}:*rbf1*^{SI} × SRZ2		521 P_{otef}:*rbf1*^{MI} × SRZ2		521		SRZ2		521 P_{otef}:*rbf1*^{SI}		521 P_{otef}:*rbf1*^{MI}	
n	135		135		135		45		45		45		45	
	DI	IR%	DI	IR%	DI	IR%	DI	IR%	DI	IR%	DI	IR%	DI	IR%
5 DPI	0.24	22.2	0.21	20.0	0.53	45.9	0.00	0.0	0.07	6.7	0.07	6.7	0.33	33.3
10 DPI	1.40	68.1	1.71	80.0	1.81	91.9	0.03	8.9	0.30	20.0	0.23	22.2	0.57	68.9

Figure S13. Hybrids constitutively expressing *rbf1* had greater area of anthocyanin induction and increased infection rates relative to the unaltered hybrid at 5 & 10 DPI. Symptoms were scored after infection of seven-day old *Z. mays* seedlings. Colored bars represent the percentage of plants that developed symptoms, as indicated in the legend. Statistical differences were calculated using Mann-Whitney U tests (**= p<0.01; **** = p<0.0001). The table presents the number of plants infected (*n*), disease index (DI), and infection rate (IR). Three separate experiments were conducted, and combined results are presented.

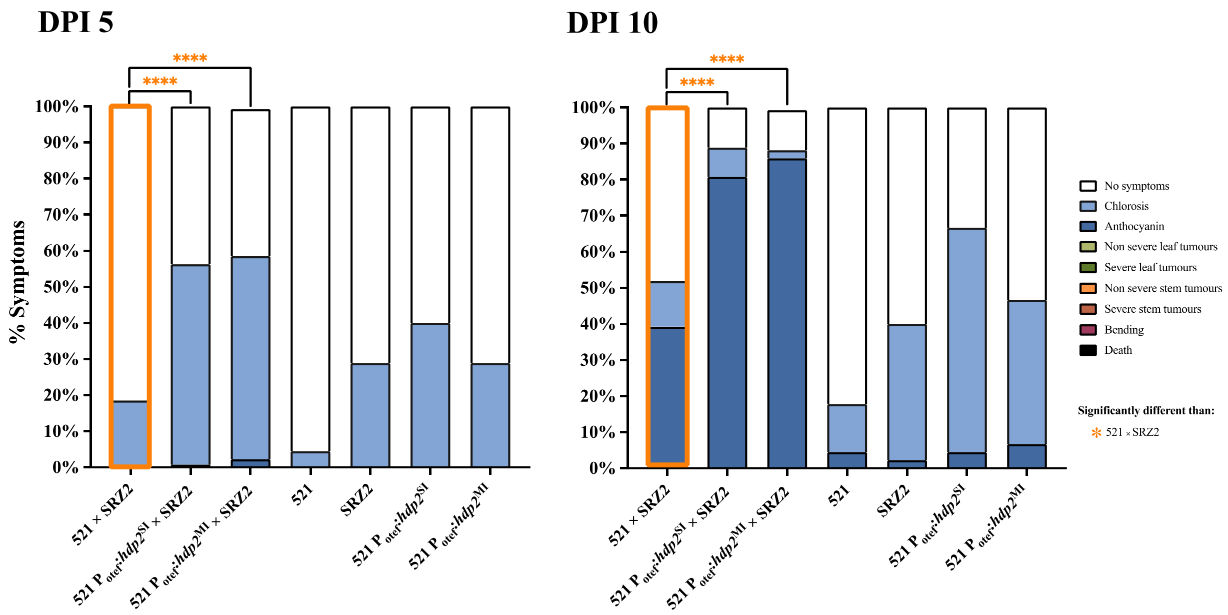

Strains	521 × SRZ2		521 Potef:*hdp2*SI × SRZ2		521 Potef:*hdp2*MI × SRZ2		521		SRZ2		521 Potef:*hdp2*SI		521 Potef:*hdp2*MI	
n	135		135		135		45		45		45		45	
	DI	IR%	DI	IR%	DI	IR%	DI	IR%	DI	IR%	DI	IR%	DI	IR%
5 DPI	0.19	18.5	0.57	56.3	0.61	59.0	0.04	4.4	0.29	28.9	0.40	40.0	0.29	28.9
10 DPI	0.91	51.9	1.70	88.9	1.75	88.8	0.22	17.8	0.42	40.0	0.71	66.7	0.53	46.7

Figure S14. Hybrids constitutively expressing *hdp2* had greater area of anthocyanin induction and increased infection rates compared to the unaltered hybrid at 5 & 10 DPI. Symptoms were scored after infection of seven-day old *Z. mays* seedlings. Colored bars represent the percentage of plants that developed symptoms, as indicated in the legend. Statistical differences were calculated using Mann-Whitney U tests (**** = $p<0.0001$). The table presents the number of plants infected (*n*), disease index (DI), and infection rate (IR). Three separate experiments were conducted, and combined results are presented.

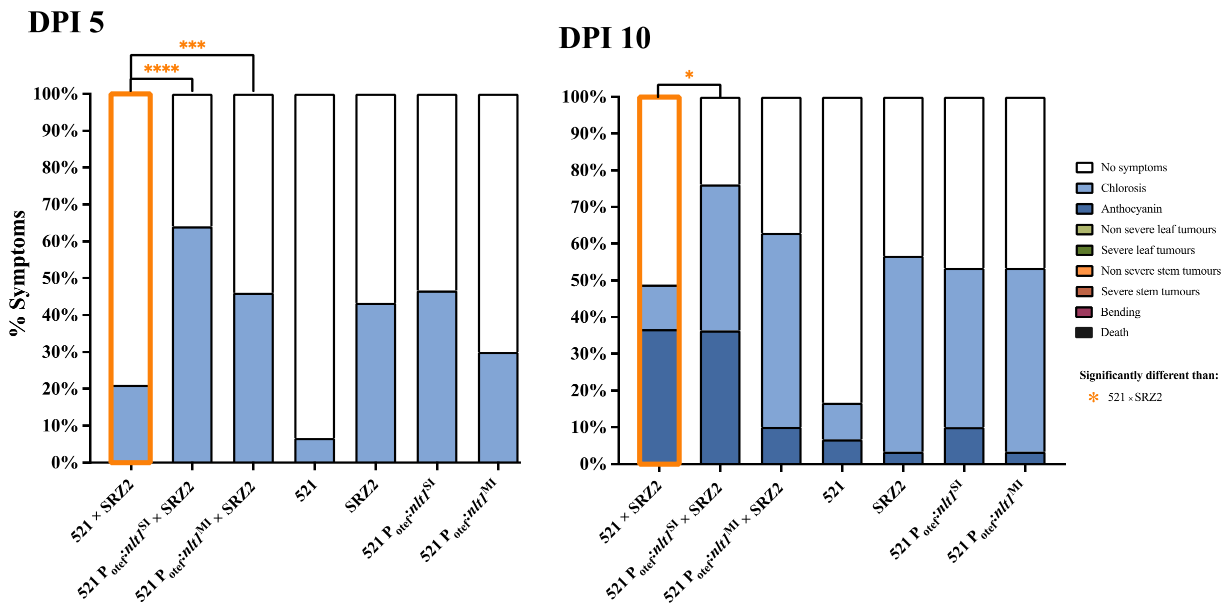

Strains	521 × SRZ2		521 Potef:*nlt1*SI × SRZ2		521 Potef:*nlt1*MI × SRZ2		521		SRZ2		521 Potef:*nlt1*SI		521 Potef:*nlt1*MI	
n	90		89		89		30		30		30		30	
	DI	IR%	DI	IR%	DI	IR%	DI	IR%	DI	IR%	DI	IR%	DI	IR%
5 DPI	0.21	21.00	0.64	64.00	0.46	46.10	0.07	6.70	0.43	43.30	0.47	46.70	0.30	30.00
10 DPI	0.86	48.90	1.13	76.10	0.73	62.90	0.23	16.70	0.60	56.70	0.63	53.30	0.57	53.30

Figure S15. Hybrids constitutively expressing *nlt1* had lower virulence than the unaltered hybrid at 5 & 10 DPI. Colored bars represent the percentage of plants that developed symptoms, as indicated in the legend. Statistical differences were calculated using Mann-Whitney U tests (*= p<0.05; ***= p<0.001; **** = p<0.0001). The table presents the number of plants infected (*n*), disease index (DI), and infection rate (IR). Two separate experiments were conducted, and combined results are presented.

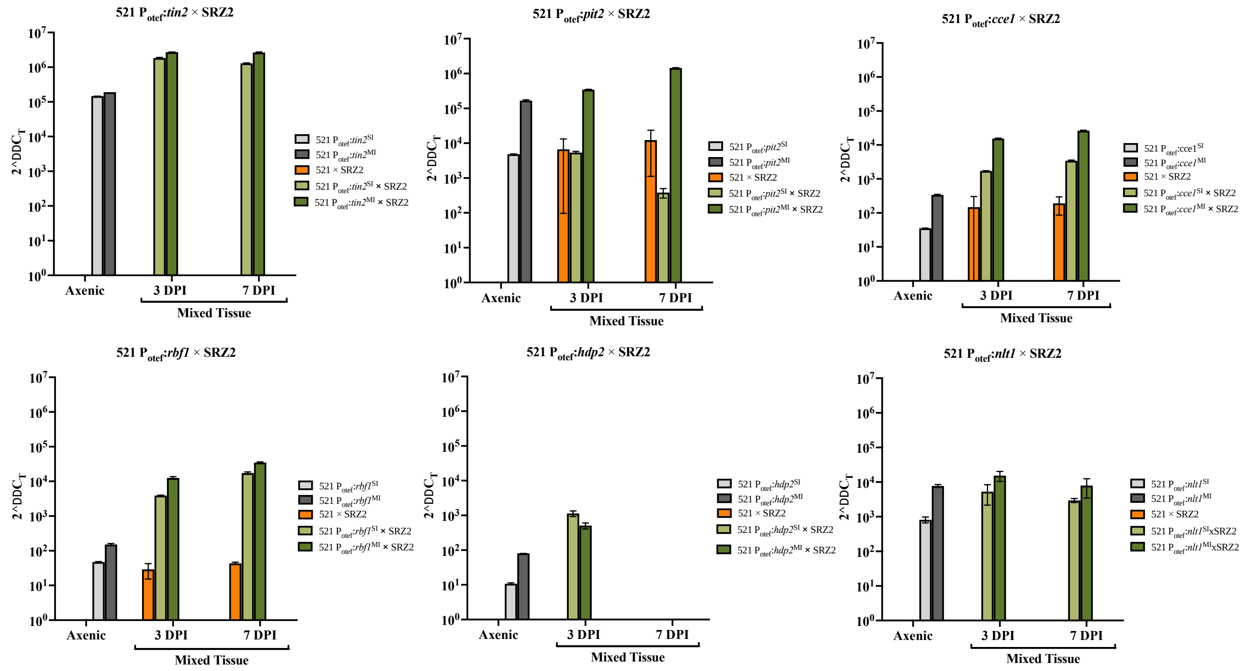

Figure S16. RT-qPCR assessment of effector and transcription factor transcript levels during infections with altered hybrids. RNA was isolated from axenically grown haploid cultures and infected plant tissue harvested at DPI 3 and 7. RT-qPCR analysis was performed using three technical replicates for each sample and the housekeeping gene (*gapdh)* transcript levels for normalization. Relative expression was determined using the $2^{-\Delta\Delta Ct}$ method with the axenically grown 521 haploid for reference. One biological replicate is reported. Transcripts from the 521 × SRZ2 infection (Figure 4) were plotted in orange to provide context.

Supplementary References

1. Heimel, K.; Scherer, M.; Vranes, M.; Wahl, R.; Pothiratana, C.; Schuler, D.; Vincon, V.; Finkernagel, F.; Flor-Parra, I.; Kämper, J. The transcription factor rbf1 is the master regulator for b-mating type controlled pathogenic development in Ustilago maydis. *PLoS Pathog.* 2010, *6*, 17–18.

2. Flor-Parra, I.; Vranes, M.; Kamper, J.; Pérez-Martín, J. Biz1, a Zinc Finger Protein Required for Plant Invasion by Ustilago maydis, Regulates the Levels of a Mitotic Cyclin. *Plant Cell* **2006**, *18*, 2369–2387, doi:10.1105/tpc.106.042754.

3. Pothiratana, C. Functional characterization of the homeodomain transcription factor Hdp1 in Ustilago maydis, 2008.

4. Lanver, D.; Berndt, P.; Tollot, M.; Naik, V.; Vranes, M.; Warmann, T.; Münch, K.; Rössel, N.; Kahmann, R. Plant Surface Cues Prime Ustilago maydis for Biotrophic Development. *PLoS Pathog.* **2014**, *10*, doi:10.1371/journal.ppat.1004272.

5. Donaldson, M.E.; Meng, S.; Gagarinova, A.; Babu, M.; Lambie, S.C.; Swiadek, A.A.; Saville, B.J. Investigating the Ustilago maydis/Zea mays pathosystem: Transcriptional responses and novel functional aspects of a fungal calcineurin regulatory B subunit. *Fungal Genet. Biol.* **2013**, *58–59*, 91–104, doi:10.1016/j.fgb.2013.08.006.

6. Lanver, D.; Müller, A.N.; Happel, P.; Schweizer, G.; Haas, F.B.; Franitza, M.; Pellegrin, C.; Reissmann, S.; Altmüller, J.; Rensing, S.A.; et al. The biotrophic development of ustilago maydis studied by RNA-seq analysis. *Plant Cell* **2018**, *30*, 300–323, doi:10.1105/tpc.17.00764.

7. Tanaka, S.; Brefort, T.; Neidig, N.; Djamei, A.; Kahnt, J.; Vermerris, W.; Koenig, S.; Feussner, K.; Feussner, I.; Kahmann, R. A secreted Ustilago maydis effector promotes virulence by targeting anthocyanin biosynthesis in maize. *Elife* **2014**, *2014*, 1–27, doi:10.7554/eLife.01355.001.

8. Mueller, A.N.; Ziemann, S.; Treitschke, S.; Aßmann, D.; Doehlemann, G. Compatibility in the Ustilago maydis-Maize Interaction Requires Inhibition of Host Cysteine Proteases by the Fungal Effector Pit2. *PLoS Pathog.* **2013**, *9*, doi:10.1371/journal.ppat.1003177.

9. Doehlemann, G.; Van Der Linde, K.; Aßmann, D.; Schwammbach, D.; Hof, A.; Mohanty, A.; Jackson, D.; Kahmann, R. Pep1, a secreted effector protein of Ustilago maydis, is required for successful invasion of plant cells. *PLoS Pathog.* 2009, *5*.

10. Seitner, D.; Uhse, S.; Gallei, M.; Djamei, A. The core effector Cce1 is required for early infection of maize by Ustilago maydis. *Mol. Plant Pathol.* **2018**, *19*, 2277–2287, doi:10.1111/mpp.12698.

11. Brachmann, A.; Schirawski, J.; Mu, P.; Kahmann, R. An unusual MAP kinase is required for efficient penetration of the plant surface by Ustilago maydis. *EMBO J.* **2003**, *22*, 2199–2210.

12. Scherer, M.; Heimel, K.; Starke, V.; Kamper, J. The Clp1 Protein Is Required for Clamp Formation and Pathogenic Development of Ustilago maydis. *Plant Cell* **2006**, *18*, 2388–2401, doi:10.1105/tpc.106.043521.

13. Heimel, K.; Scherer, M.; Schuler, D.; Kamper, J. The Ustilago maydis Clp1 Protein Orchestrates Pheromone and b-Dependent Signaling Pathways to Coordinate the Cell Cycle and Pathogenic Development. *Plant Cell* **2010**, *22*, 2908–2922, doi:10.1105/tpc.110.076265.
